# The Role of PET/CT Molecular Imaging in the Diagnosis of Recurrence and Surveillance of Patients Treated for Non-Small Cell Lung Cancer

**DOI:** 10.3390/diagnostics6040036

**Published:** 2016-09-30

**Authors:** Julio Francisco Jiménez-Bonilla, Remedios Quirce, I. Martínez-Rodríguez, María De Arcocha-Torres, José Manuel Carril, Ignacio Banzo

**Affiliations:** Nuclear Medicine Department, University Hospital Marqués de Valdecilla, Molecular Imaging IDIVAL, University of Cantabria, 39008 Santander, Spain; mnuqpm@humv.es (R.Q.); mimartinez@humv.es (I.M.-R.); marcocha@humv.es (M.D.A.-T.); manuel.carril@unican.es (J.M.C.); mnubmj@humv.es (I.B.)

**Keywords:** non-small lung cancer, recurrence, ^18^F-FDG, PET/CT, surveillance, follow-up

## Abstract

Non-small cell lung cancer (NSCLC) is the leading cause of cancer mortality worldwide and its prognosis remains poor. Molecular imaging with ^18^F-FDG PET/CT can metabolically characterize the nature of lesions as benign or malignant, allowing a better staging at the diagnosis of this kind of patient. This advantage can also be applied in the re-staging due to the suspicion of recurrent disease. Many patients have a recurrence of the disease, including surgically treated patients. In the current context, with new personalized oncological treatments, the surveillance for recurrence and its accurate diagnosis are crucial to improve their survival. In this paper, we revise the current knowledge about the clinical and molecular factors related to the recurrent disease. In the context of new, promising, available personalized treatments, the role of molecular imaging with PET/CT and ^18^F-FDG and non-^18^F-FDG radiotracers in the follow-up of NSCLC-treated patients is especially attractive and interesting.

## 1. Introduction

Non-small cell lung cancer (NSCLC) is the neoplasm responsible for higher mortality in the Western world (27%) and still has a poor prognosis, with an overall survival at five years around 15%. Unfortunately, in most patients (80%), the disease is diagnosed at an advanced stage (III**–**IV) and less in the early stages (I**–**II), when it would be potentially curable. Early diagnosis and accurate staging are key to successful treatment. Molecular imaging with ^18^F-FDG PET/CT provides metabolic information, which allows a better differentiation of benign and malignant tissue, revealing the functional abnormalities that precede structural damage. Currently, it is routinely used for staging patients and its introduction has an impact as high as 30% for a change in the staging made with conventional imaging techniques. This fact contributed to a better selection for candidates for radical treatments with a curative purpose, such as surgery or stereotactic radiotherapy (SSRT) [1].

Recurrence is a common phenomenon in the natural history of the disease, which occurs in a variable proportion of patients (20%–80%) and determines their survival [2]. The knowledge of the pathophysiologic mechanisms has been the focus of a growing number of works from the past decade, which is explained because they have also expanded the therapeutic options. Moreover, the PET/CT image has been successfully applied in the diagnosis of disease recurrence and has an advantage over other imaging techniques, with the same basis as the staging (restaging) [3]. However, PET/CT examination is not recommended in the guidelines for the monitoring and surveillance of NSCLC patients. In this paper, we review the current state of knowledge of the factors linked to its recurrence, the recurrence patterns and the role of molecular imaging with PET/CT in the era of new personalized cancer treatments and their potential impact on the survival of patients.

## 2. Factors and Patterns of Recurrence of Non-Small Cell Lung Cancer

NSCLC recurrence is frequent in the natural history of the disease. The recurrence is considered loco-regional when it is limited to the hemithorax where the primary tumor was located and includes the ipsilateral lymph nodes, bronquial stump, pleura and chest wall. The most common sites for distant recurrence are reported on the same sites where metastases were found at staging [4]. Until now, the most important group to be cured is the patients in an early stage in whom a complete surgical resection of the primary tumor can be obtained. Unfortunately, 30%–55% of patients with NSCLC develop a recurrence and die of their disease despite curative resection [5]. Post-surgery recurrence has been summarized by two reasons: an underestimation of the true tumor stage, probably due to occult micro-metastases cancer cells, and, second, because the removal of the tumor during surgery itself might lead to the dissemination of cancer cells [6]. However, in a deeper analysis, Boyd et al. examined the timing of the local and distant failure of 250 patients who developed recurrent disease among 975 patients undergoing surgery [7]. They found that 17%, 44% and 39% of the recurrences were confined to local only, distant only, and both, respectively. Only 0.6% of the patients developed a sequential local recurrence, suggesting that the crude local recurrence rate might be underestimated if only the first sites of failure are scored. Distant metastases and local recurrence might arise from occult cancer cells or due to dissemination during surgery, as has previously been suggested.

The classic classification of malignant tumours (TNM) staging shows several limitations as it is only based on clinical and pathological findings [8], and a group of different factors related to recurrence following surgery are summarized in Table 1. In the new era of personalized medicine, these factors should be taken into account [6,9].

A temporal pattern in the diagnosis of NSCLC recurrence has been observed. The median time from surgery to relapse is 13.9 months for local failure and 12.5 months for distant failure [7]. Moreover, Watanabe et al., in an interesting paper [10], note that there are different recurrence peaks during post-operative follow-up. Indeed, a structured multipeak pattern of recurrence risk has been described, with a bimodal recurrence pattern similar to that in patients with breast cancer. One was found at one year after surgery, suggesting that surgical invasion disrupts homeostasis, accelerating the proliferation of dormant cancer cells, and another peak was noted at the end of the second year of follow-up. A small peak was found even five years after surgery. The second and subsequent peaks of recurrence described in this study could be explained, according to the authors, by the hypothesis that residual tumor cells proliferated and micro-metastases developed after entering a transient state of dormancy. However, the underlying mechanisms remain to be fully clarified.

On the other hand, the recurrence dynamic of lung cancer with the bimodal characteristic pattern seems to also be sex-dependent, with differences in the first peak (men at six to eight months after surgery and women one year after) and a tendency to increase at two years in women. From a histological point of view, the recurrence due to adenocarcinoma seems to appear later than squamous cell carcinoma recurrences.

Finally, several metabolic studies assessing the intensity of the glucose metabolism in primary tumors of NSCLC have been conducted, and a high standardized uptake value (SUV) of tumors has been related to an increased risk of recurrence. However, prospective and well-designed studies are necessary to obtain a personalized prediction for each patient and to plan the follow-up [11].

## 3. The Diagnosis of NSCLC Recurrence

The contribution of ^18^F-FDG PET/CT to the staging of patients diagnosed of NSCLC is well known and is considered the current clinical gold standard imaging modality for this purpose. Moreover, it was pointed out as superior to standard CT and MRI for the detection of disease after surgery and to local ablative therapies such as microwave ablation and chemotherapy [12]. In a preoperative staging of NSCLC, as Fisher et al. have pointed, the use of PET-CT can reduce both the total number of thoracotomies and the number of futile thoracotomies with no effect (negative or positive) on overall survival [13]. Furthermore, in patients without enlarged lymph nodes and a PET-negative mediastinum, these authors suggest that the patient may proceed directly to surgery. However, enlarged lymph nodes on CT need confirmation independently of PET findings and a positive finding on PET/CT needs confirmation before a decision on surgery is made [14].

**Figure 1:**
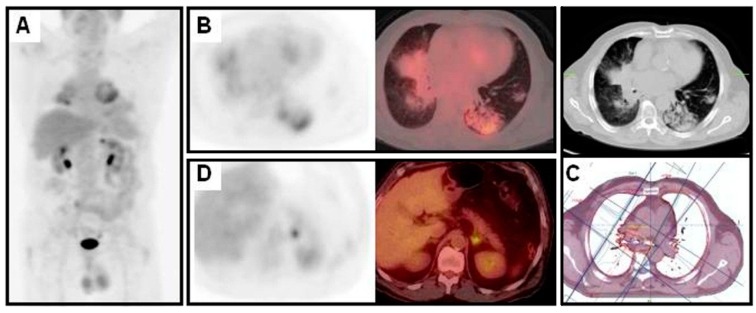
Patient with an adenocarcinoma near the right hilum treated with chemo-radiotherapy. A follow up CT performed four months later showed non-conclusive radiological lung findings. An ^18^F-FDG PET/CT showed increased lung uptake related to the radiotherapy area (**A**–**C**); Also, focal hypermetabolism in left adrenal gland was detected (**D**). Surgery confirmed an adrenal metastasis.

**Figure 2:**
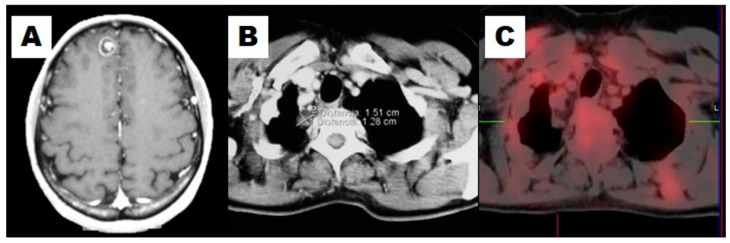
This example illustrates how ^18^F-FDG PET/CT would rule out the suspicion of recurrence. A resectable isolated cerebral lesion was diagnosed in a 48-year-old female after treatment of NSCLC (**A**); She had a previous lobectomy and a left adrenalectomy. Taking into account the resectability of the cerebral lesion, a CT for restaging showed a solitary nodule in the right upper lung (**B**); An ^18^F-FDG PET/CT showed no pathological uptake in the lung nodule (**C**). The patient was treated with stereotaxic surgery over the cerebral lesion. Three years later, she remained asymptomatic.

**Figure 3:**
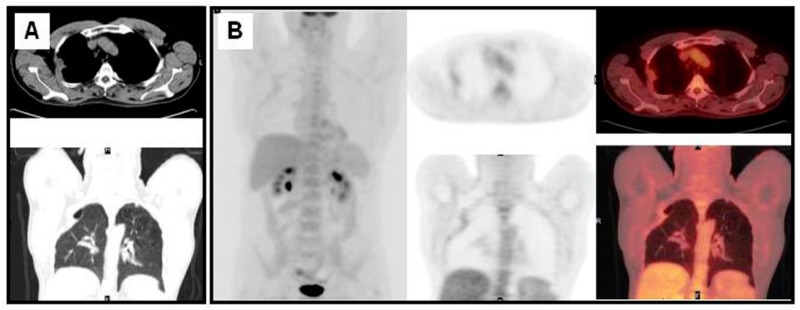
Patient with undifferentiated carcinoma of the right lung surgically treated seven years before followed by radiotherapy. In a context of pain and functional impotence of the right shoulder, a CT showed a suspicious image in the medial aspect of the lung near the right axilla and structural changes due to the previous surgery (**A**); ^18^F-FDG PET/CT ruled out recurrence (**B**).

**Figure 4:**
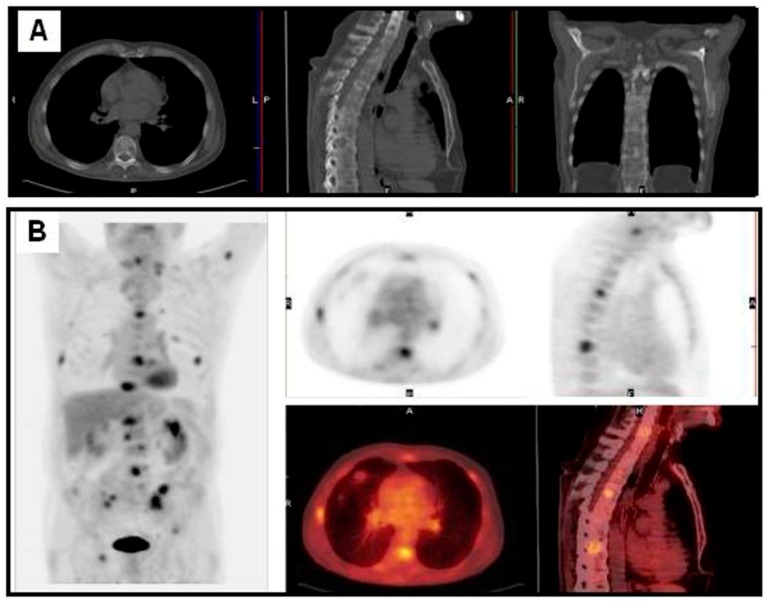
Patient with a NSCLC in the left superior lobe treated three years before with chemo-radiotherapy. A radiological exam showed sclerotic bone lesions (**A**); FDG PET/CT showed multiple bone, left supraclavicular lymph nodes and lung metastases (**B**).

^18^F-FDG PET/CT is used to evaluate equivocal CT findings, due to its very high accuracy for distinguishing recurrent disease from benign post-treatment changes (Figure 1). If tumor recurrence is suspected or confirmed, ^18^F-FDG-PET/CT for restaging is indicated to differentiate local from distant recurrence and, thus, to plan local or systemic treatment [15] (Figure 2, Figure 3 and Figure 4). In our experience, the sensitivity and specificity of ^18^F-FDG PET/CT were found as high as 100% and 78%, respectively, and this diagnostic advantage is likely related to its superiority in the staging of NSCLC patients [15]. In this sense, a meta-analysis of ^18^F-FDG-PET/CT and CT in the mediastinal staging of NSCLC demonstrated an accuracy of 86% for ^18^F-FDG-PET/CT and of 73% for CT. Sensitivity, specificity, positive and negative predictive values were 73%, 91%, 71%, and 90% for ^18^F-FDG-PET/CT and 74%, 73%, 52%, and 88% for CT, respectively. The ability to detect distant metastases with a high sensitivity, specificity, and accuracy (94%, 97%, and 96%, respectively) renders PET/CT superior to other imaging modalities. An additional magnetic resonance imaging (MRI) study of the brain is recommended in patients with stage II**–**IV disease for complete accurate whole-body tumor staging [16].

Beyond ^18^F-FDG, other radiotracers such as [18F]-fluoro-3′-deoxy-3′-l-fluorothymidine (^18^F-FLT), which mainly reflects cellular proliferation, are being evaluated to assess the real contribution to improving the clinical outcome of NSCLC patients. Most of the works have been done to evaluate the treatment response in cancer patients [17], but their use for the diagnosis of recurrence remains open. In the selected context of pulmonary neuroendocrine tumors, ^68^Ga-DOTATATE PET seems to have better results for staging and response evaluation than ^18^F-FDG PET/CT and to evaluate negative or equivocal findings on ^111^In-DTPA-Octreotide scintigraphy [18].

At present, other imaging modalities have been analyzed. In this context, promising results have been reported with dynamic contrast-enhancement CT (DCT-CT), but this technique does not seem ready for clinical routine use yet [12]. Multi-parametric magnetic resonance imaging (MRI) is the gold standard for the assessment of brain metastases [19]. MRI has the best diagnostic capabilities to visualize mediastinal and chest wall infiltration due to both inherent high soft-tissue contrast and its ability to acquire dynamic cine-MRI sequences, minimizing artifacts from cardiac and respiratory motion. Moreover, it is useful for bone metastasis, but its diagnostic accuracy when compared with ^18^F-FDG PET/CT and bone scintigraphy is equal or slightly less [20]. PET/MRI has been introduced and applied recently in a few Diagnostic Centers. The potential advantage of this technique is based on the combination of metabolic/functional PET information, high soft-tissue contrast of MRI and DW-MRI; however, the current literature is insufficient to establish a clear role of PET/MRI for lung cancer imaging.

## 4. The Surveillance of NSCLC Recurrence

The high incidence of recurrence of the disease during the first two years after NSCLC treatment is well known and is the reason a special follow-up is needed. However, there is no consensus about what the best is strategy for this purpose. The American College of Chest Physicians (ACCP) guidelines recommend surveillance by clinical examination and chest radiography or CT every six months for two years and then yearly for patients with good performance status and pulmonary function [21]. The National Comprehensive Network Cancer (NCNC) recommends a history and physical examination with contrast-enhanced CT every four to six months for two years and then a physical examination and non-contrast-enhanced CT annually; the European Society for Medical Oncology (ESMO) note that there is no clear role for routine studies in asymptomatic patients and in patients in whom no intervention is planned [22].

In summary, PET and brain MRI are not currently recommended for routine follow-up. However, ^18^F-FDG PET/CT has shown a better diagnostic accuracy than diagnostic CT to assess the suspicion of NSCLC recurrence [15,23,24].

Recently, Marcus et al. [25] have approached the open question about why there have been many studies demonstrating the impact of ^18^F-FDG PET/CT on the management plan at staging and in patients with suspected recurrent disease, and there is no substantial literature evaluating this clinical question in the follow-up PET/CT studies. Unlike other works, these authors noted that PET/CT identified recurrence in 44.3% of scans performed without prior clinical suspicion of recurrence and ruled out recurrence in 24.2% of scans performed with prior clinical suspicion of recurrence. They conclude that ^18^F-FDG PET/CT adds value to the clinical assessment when it is performed in the absence of prior suspicion of recurrence and has a clinical impact on the management of the patients as high as 28.1%. There are several limitations derived from the aim of the study, but the results are interesting.

The contradictory results of other studies that did not find a significant increment of global survival when ^18^F-FDG PET/CT was routinely applied in the surveillance of NSCLC patients could be discouraging for this technique. However, this fact can indicate that the surveillance of each patient should be planned on the basis of their individual characteristics and not generalized from a global recommendation.

At this moment, two different questions could be approached: what new contributions on risk factors for recurrence have become known in the last years to make a personalized surveillance for patients treated for NSCLC, and should ^18^F-FDG PET/CT be applied routinely, or only for patients in whom recurrent disease is suspected?

First, the metabolism data of the primary tumor before surgical or radiation treatment has been related to the time free of recurrence (TFR). High values of SUVmax, metabolic tumor volume (MTV) and total lesion glycolysis (TLG) predicted a higher risk of recurrence or death in patients treated surgically, as is noted in a recent meta-analysis [9], suggesting that the use of ^18^F-FDG PET/CT may benefit the selection of patients for more aggressive treatments. Besides, a clinical trial about an adaptive neoadjuvant chemotherapy guided by ^18^F-FDG PET/CT in resectable NSCLC [26] showed the utility of this technique to assess the response and then change the chemotherapy regimen in non-responding patients. They suggest that this adaptive approach can be also used to test new drugs, attempting to optimize perioperative chemotherapy to achieve better long-term outcomes. On the other hand, Ito et al. [27] have shown the correlation between SUVmax and tumor invasiveness or post-surgical recurrence of solid types of NSCLC, and in particular, in solid-type adenocarcinoma, it was correlated with recurrence. The age of the patients does not seem to be a factor in predicting recurrence after resection [28]. The nodal stage of surgically resected NSCLC has been directly associated with distant recurrence and overall survival but not with loco-regional recurrence [29]. In a study, the cut-off SUV value of 4.5 was determined by the receiver operating characteristic (ROC) curves of all patients, and the same cut-off value was applied to both adenocarcinoma and squamous cell carcinoma. In the adenocarcinoma group (*n* = 158), a PET SUV ≥2 was significantly associated with five-year recurrence using the Kaplan-Meier with log-rank test. No one has shown recurrence in patients with SUV <2. However, in the squamous cell carcinoma group, there was no significant association between a high level of PET SUV and tumor recurrence with a cut-off value of 9 or 10 [30].

More relevant is the growing evidence of the importance of epidermal growth factor receptor status (EGFR) as a factor for post-recurrence survival in surgically treated patients. Kudo et al. reported that the EGFR and pathological stage are related, with a better survival after disease recurrence [31]. Glubb et al. suggested that FLT1 genetic variation may be a prognostic factor for recurrence in stages I**–**III, and should be tested in the adjuvant setting of NSCLC [32]. The correlation between EGFR mutation status and FDG uptake has not been well-established. A recent report from a global point of view has pointed out that EGFR mutation–positive NSCLC patients have relatively lower glycolysis compared with NSCLC patients without EGFR mutation (SUVmax 7.0 ± 3.9 vs. 10.3 ± 5.8) [33]. In addition, it has been observed that NSCLC patients with tumors harboring a K-RAS mutation showed significantly higher ^18^F-FDG uptake (SUVmean 9.5) than wild-type K-RAS and a multivariate model based on age, gender, stage and SUVmean might be used as a predictive marker of K-RAS mutation status in patients with stage III or IV NSCLC [34].

Second, the other important question is how to design the surveillance of NSCLC patients. Takenaka et al., in a prospective work, compared the assessment of recurrence in a population of 92 consecutive surgically treated patients of NSCLC in whom the follow-up was made with radiological standard examinations and whole-body ^18^F-FDG PET/CT [35]. There were no statistically significant differences when ROC curves were used to compare the diagnostic yield of both methods of surveillance. Sudarski et al. underlined that this factor might play a crucial role for an efficient workflow of a large department that follows up large patient cohorts [36] and other authors have found similar results [37]. Those papers analyzed the systematic follow-up, including asymptomatic patients and patients in whom recurrence was suspected. This question of if the ^18^F-FDG PET/CT should be included routinely for all patients or only for patients with suspicion of recurrent diseases is still debatable, but there is enough evidence to support that when a recurrence is suspected, ^18^F-FDG PET/CT has better diagnostic accuracy for the detection of extra-cranial recurrence [15,22].

The follow-up of non-surgically treated patients is another point of interest. Overall, the SSRT has acceptable results and, in those patients, the surveillance of loco-regional recurrence is crucial. Indeed, a recent study showed that high ^18^F-FDG uptake on pre-radiotherapy PET/CT can identify preferential sites of local relapse after chemo-radiotherapy for NSCLC [38]. The post-therapeutic changes may make the evaluation of this kind of patient difficult, and the assessment of images must be careful, including potentially delayed and dual-point exams. The metabolic information provided by ^18^F-FDG PET/CT could resolve some difficulties inherent for conventional CT, but more studies are needed. With regard to radio-frequency therapy, more evidence is still needed to determine a SUVmax cut-off for the diagnosis of loco-regional recurrence, where PET can be indicated [39].

In conclusion, molecular imaging with ^18^F-FDG PET/CT should be considered for the restaging of NSCLC patients when recurrence is suspected. Metabolic information provided by ^18^F-FDG PET/CT can allow the selection of the most appropriate therapy. High quality evidence is still necessary regarding if the intensive follow-up strategies lead to improved survival and how PET/CT may play a role in those personalized programs of surveillance.

## 5. Future Perspectives

^18^F-FDG PET/CT has shown its value in detecting tumor-related changes. However, the presence of inflammatory abnormalities may reduce the specificity. In such a context, ^18^F-FLT PET/CT has proved to be a more sensitivity for early treatment response evaluation [40]. Although it is currently unclear whether these changes predict the eventual clinical outcome, its potential utility to clarify recurrent disease when it is strongly suspected can be considered.

According to other authors (Yano et al., 2014), the therapeutic strategy for treating post-operative recurrence in NSCLC patients should be considered according to the mode of first recurrence, taking into account that limited distant metastasis (oligo-metastasis) can be now successfully treated, knowing the ability of PET/CT to detect distant metastases and local recurrence [41].

All available resources and knowledge should be directed to prolong post-recurrence survival in patients with a good performance status. In this sense, the metabolic information provided by ^18^F-FDG and other PET/CT radiotracers may play a pivotal role, and would make a relevant contribution in this new era of personalized medicine to get the best surveillance for NSCLC patients.

## Figures and Tables

**Table 1:** Predictors for recurrence of non-small cell lung cancer after complete resection [6,9].

Clinical Parameters				
Lymphatic permeation		Pleural Invasion	Vessel invasion	
Intratumoral vascular invasion		Nodal involvement	Incomplete MLNs dissection	
Biochemical and Molecular Parameters				
High CEA MIB-1 expression MACC1 expression CK19 mRNA IGF1R	Histological Dedifferentiation Methylation (promoter regions p16 and CDH13) CXCR7 expression MicroRNA expression			KRAS Ki-67 TS expression EGFR mutations
^18^F-FDG PET or PET/CT findings in primary tumour Intensity of uptake (SUVm) Metabolic tumor volume (MTV)				

NSCLC: Non small cell lung cancer; MLNs:Mediastinal lymph nodes; SUV: Standardized uptake value; TS: Thymidylate synthase; CEA: Carcinoembryonic antigen; EGFR: Epidermal growth factor receptor; ^18^F-FDG: ^18^fluorine-fluoro deoxiglucose; PET: Positron emission tomography; CT: Computed tomography.
